# *H19* non coding RNA-derived miR-675 enhances tumorigenesis and metastasis of breast cancer cells by downregulating c-Cbl and Cbl-b

**DOI:** 10.18632/oncotarget.4976

**Published:** 2015-07-22

**Authors:** Constance Vennin, Nathalie Spruyt, Fatima Dahmani, Sylvain Julien, François Bertucci, Pascal Finetti, Thierry Chassat, Roland P. Bourette, Xuefen Le Bourhis, Eric Adriaenssens

**Affiliations:** ^1^ INSERM U908, Cell Plasticity and Cancer, F-59655, Villeneuve d'Ascq, France; ^2^ University of Lille, F-59655, Villeneuve d'Ascq, France; ^3^ CNRS UMR 8161, F-59021, Lille, France; ^4^ Paoli-Calmettes Institute, Aix -Marseille University, F-13009, Marseille, France; ^5^ PLETHA, Institut Pasteur Lille, F-59019, Lille, France

**Keywords:** H19, miRNA, breast cancer, CBL, tyrosine kinase receptor

## Abstract

*H19* is a long non-coding RNA precursor of miR-675microRNA. *H19* is increasingly described to play key roles in the progression and metastasis of cancers from different tissue origins. We have previously shown that the *H19* gene is activated by growth factors and increases breast cancer cell invasion. In this study, we established *H19*/miR-675 ectopic expression models of MDA-MB-231 breast cancer cells to further investigate the underlying mechanisms of *H19* oncogenic action. We showed that overexpression of *H19*/miR-675 enhanced the aggressive phenotype of breast cancer cells including increased cell proliferation and migration *in vitro*, and increased tumor growth and metastasis *in vivo*. Moreover, we identified ubiquitin ligase E3 family (c-Cbl and Cbl-b) as direct targets of miR-675 in breast cancer cells. Using a luciferase assay, we demonstrated that *H19*, through its microRNA, decreased both c-Cbl and Cbl-b expression in all breast cancer cell lines tested. Thus, by directly binding c-Cbl and Cbl-b mRNA, miR-675 increased the stability and the activation of EGFR and c-Met, leading to sustained activation of Akt and Erk as well as enhanced cell proliferation and migration. Our data describe a novel mechanism of protumoral action of *H19* in breast cancer.

## INTRODUCTION

The *H19* gene, located in human in 11p15.5 locus, is submitted to genomic imprinting. It is expressed only from the maternal allele [1]. It is transcribed by the RNA polymerase II and the transcript is spliced, polyadenylated, capped and exported into the cytosol. However, no protein associated to this transcript has been discovered and Brannan et al. proposed that *H19* RNA functions as a riboregulator [2]. *H19* is highly expressed in the extraembryonic tissues (placenta), the embryo proper, and the fetal tissues. After birth, its expression is repressed even if a basal expression subsists in several tissues including mammary gland, adrenal gland and uterus [3–6]

The role of *H19* in cancer is still matter of debate. It has been proposed that *H19* acts as a tumor suppressor in Wilm's tumors, embryonic rhabdomyosarcoma, and the Beckwith-Wiedemann syndrome [7]. In addition, using *in vivo* mice models of tumorigenesis, a role of tumor suppressor gene has been ascribed to *H19* [8]. However, numerous studies have shown that *H19* is an oncogene in many types of cancers. Indeed, *H19* overexpression is often correlated with poor prognosis in bladder, lung, oesophageal and gastric cancers [9–13]. *H19* exerts its oncogenic activity through different mechanisms. For example, it has been reported that *H19* functions as a Myc-up-regulated gene to potentiate the tumorigenic phenotype of breast and lung cancer cells [14]. More recently, *H19* was described to act as a molecular sponge to regulate the let-7 family of miRNAs [15]. In addition, *H19* is also a precursor for microRNA-675 (miR-675) and generates two mature miRNAs, miR-675-5p (miR-675) and miR-675-3p (miR-675*) [16].

MicroRNAs (miRNAs) are 19- to 25-nucleotide regulatory non-coding RNAs that are initially expressed as hairpin transcripts of primary miRNA under the control of RNA polymerase II. These primary miRNA hairpins are cleaved by two enzymes, Drosha and Dicer, to generate mature miRNAs. Although several mechanisms of gene expression regulation by miRNAs have been demonstrated [17], they mainly repress gene expression at the post-transciptional level by interacting with 3′UTR of target mRNA.

Recent data indicate that *H19*-derived miR-675 favours tumor progression by repressing the expression of several target genes, including *Rb* in colorectal cancer [12], *Twist1* in hepatocellular carcinoma [18]*, and RUNX1* in gastric cancer [19].

We have previously shown that *H19* is overexpressed in 70% of breast cancer [3]. *H19* gene overexpression in mammary epithelial cells promotes tumorigenesis by upregulating thioredoxin, a modulator of signal transduction and potentiator of tumorigenesis [20]. *H19* gene is up-regulated by growth factors such as HGF and by transcription factors such as E2F1 to enhance cell invasion and cell cycle progression [21, 22]. Altogether theses finding are in favor of a role of *H19* as an oncogene in breast cancer [23].

In this study, we have examined the role of *H19*-derived miR-675 in controlling the properties of breast cancer cells. Using *in silico* prediction and functional assays, we identified c-Cbl and Cbl-b as direct targets of miR-675. *H19*-miR-675-Cbl increased the expression level of tyrosine kinase receptors and sustained their activation of down-stream signaling pathways. Moreover, miR-675 overexpression increased the aggressive phenotype of breast cancer cells both *in vitro* and *in vivo*. Our findings provide novel mechanistic insights into a critical role for *H19* RNA in breast cancer development and reveal a previously unknown link between *H19*/miR-675, Cbl and tyrosine kinase receptors to enhance breast cancer cell aggressiveness.

## RESULTS

### *H19*-derived miR-675 targets c-Cbl and Cbl-b through their coding sequences in breast cancer cells

**Figure 1:**
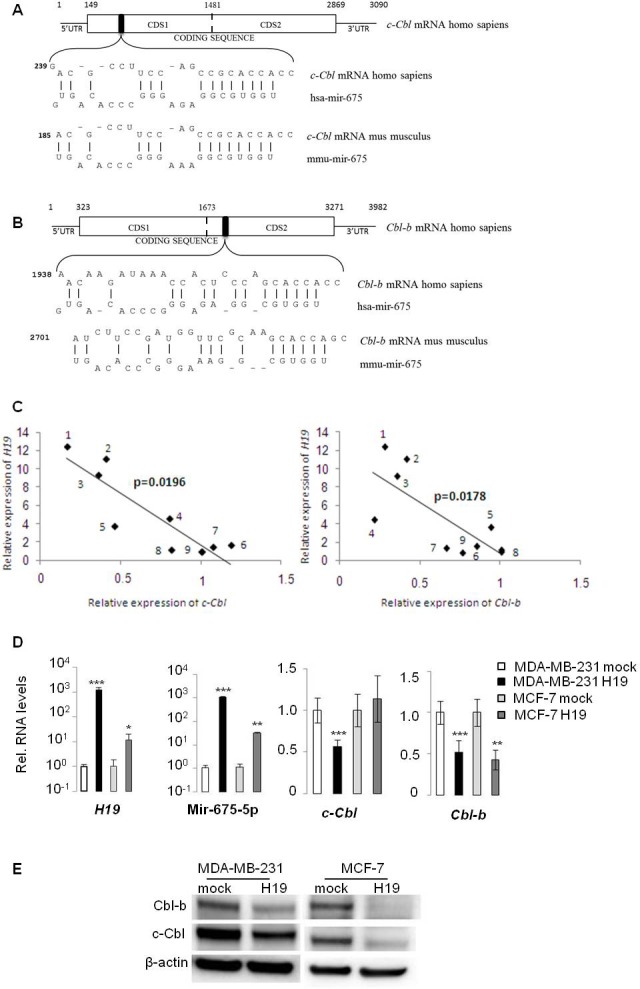
*H19/miR675* downregulated c-Cbl and Cbl-b expression in breast cancer cells **A.**, **B.** Alignment prediction of miR-675-5p on *c-Cbl* and *Cbl-b* mRNA. Relative positions are indicated in bp. Note interaction of miR-675 on *Cbl* mRNA is conserved in human and mouse. Coding sequences of theses mRNA are too long, so we cloned them in pMiR-REPORT luciferase in two parts named CDS1 and CDS2. The artificial break is represented by the dotted line. **C.** Negative correlation between *H19* and c-Cbl/Cbl-b expression in breast cancer cell lines [24]. Relative expression of *H19* and *c-Cbl* or *Cbl-b* in (1) MDA-MB-361, (2) MDA-MB-134, (3) SUM225, (4) T47D, (5) S68, (6) SUM159, (7) MCF-7, (8) ZR-75-30 and (9) BT483. **D.** QRT-PCR analysis of expression of *H19*, miR-675-5p, *c-Cbl* and *Cbl-b* in breast cancer cell lines. Results are presented as relative levels compared to MDA-MB-231 mock cells (indexed to 1). Data represent mean of three independent experiments and error bar sem.*p < 0.05; **p < 0.005; ***p < 0.001. **E.** Western blot analysis of c-Cbl and Cbl-b levels in MDA-MB-231 and MCF-7 breast cancer cells. β actin was used as a loading control.

We have previously demonstrated the oncogenic role of *H19* gene in breast tumorigenesis [20]. *H19* is a precursor of miR-675-5p/miR-675-3p [16], and *H19*-derived miR-675 has been reported to promote tumorigenesis of several cancers including colon and gastric cancers [12, 19]. To investigate the molecular mechanism of oncogenic miR-675 in breast cancer cells, we performed alignment prediction and found that miR-675-5p was aligned with coding sequences of 2 proteins belonging to the ubiquitin ligase E3 protein family: c-Cbl and Cbl-b (Figure 1A and 1B). Interestingly, putative seeds are located on coding sequence of these two mRNAs and are conserved between human and mouse. Furthermore, analyses of *H19* and *Cbl* family gene expression in breast cancer cell lines [24] showed a negative correlation between *H19* and c-Cbl or Cbl-b (Figure 1C). We then verified the expression of miR-675-5p and c-Cbl/Cbl-b in breast cancer cells overexpressing *H19*. As shown in Figure 1D, enhanced *H19* expression in MDA-MB-231 and MCF-7 breast cancer cell lines was correlated with an increased level of miR-675-5p. Moreover, the levels of c-Cbl and Cbl-b expression decreased significantly in *H19*-overexpressing cells (Figure 1D and 1E). Together, these data indicate that c-Cbl and Cbl-b may be negatively regulated by *H19* in breast cancer cells.

**Figure 2:**
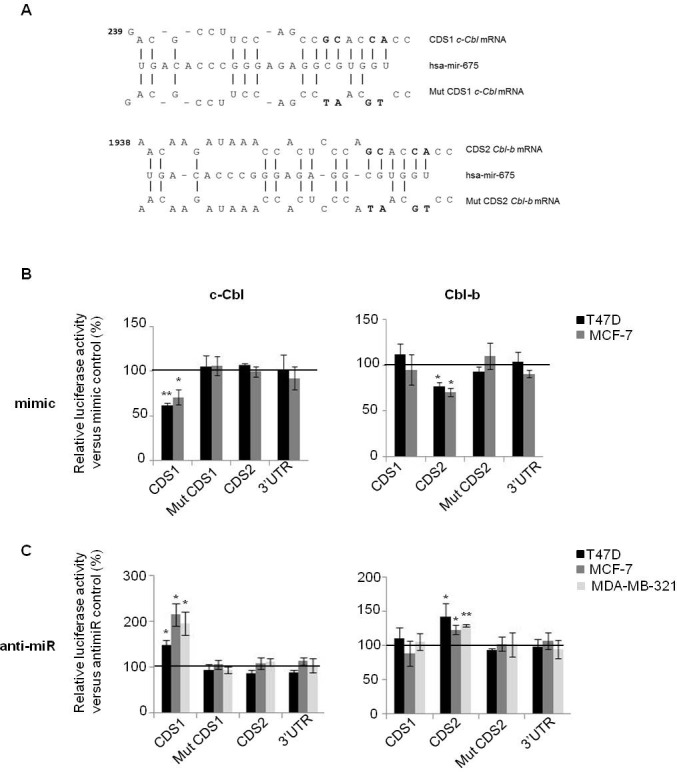
MiR-675 directly targeted on c-Cbl and Cbl-b through interaction with coding sequences **A.** Alignment prediction of miR-675-5p on *c-Cbl* and *Cbl-b* mRNA. Mutation in seed sequence is represented in bold. Coding sequence (CDS) and 3′UTR of *c-Cbl* and *Cbl-b* mRNA were cloned into pMIR-REPORT luciferase. Coding sequence of theses mRNA are so long that we cloned them in pMIR-REPORT luciferase in two parts named CDS1 and CDS2. **B.** and **C.** The firefly luciferase activity in breast cancer cells after cotransfection with reporter construct and miR-675 mimic or miR-675 inhibitor (αmir). The luciferase activity was measured by dual-luciferase reporter assay (Promega) and was normalized to Renilla luciferase activity. Plasmids were transfected with mimic or antimir, or theirs controls in breast cancer cell lines (T47D, MCF-7 and MDA-MB-231). Data represent mean of three independent experiments *versus* their respective controls in percentage and error bar sem.*p < 0.05; **p < 0.005.

To confirm the direct regulation of c-Cbl and Cbl-b by *H19*, we cloned coding sequences (CDS) of c-Cbl and Cbl-b mRNAs, each in two fragments (CDS1 and CDS2) into the *Firefly* luciferase reporter vector (pMIR-REPORT) (Figure 1A, 1B). We also cloned CDS mutated on seed sequence and 3′UTR of these mRNAs in the same vector. The mutation is represented on Figure 2A. Each of these DNA constructs were transfected into various breast cancer cells together with either miR-675 mimic, anti-miR-675 (miR-675 inhibitor) or their corresponding controls. As shown in Figure 2B (left panel), miR-675 mimic decreased relative luciferase activity of pMIR-CDS1 c-Cbl of 35-40% compared to microRNA control in MCF-7 and T47D cells. This effect was abolished when seed sequence was mutated. Furthermore, miR-675 had no effect on luciferase activity of pMIR-CDS2 c-Cbl or pMIR-3′UTR c-Cbl plasmids (Figure 2B, left panel). Similar results were obtained for Cbl-b, (Figure 2B, right panel). MiR-675 mimic decreased relative luciferase activity of pMIR-CDS2 Cbl-b of 30% in the two tested cell lines. The effect of miR-675 mimic on CDS2 was abolished after mutation in seed sequence (mut CDS2). MiR-675 mimic had no effect on luciferase activity of pMIR-CDS1 Cbl-b or pMIR-3′UTR Cbl-b plasmids (Figure 2B, right panel).

Contrary to miR-675 mimic, the miR-675 inhibitor was found to increase relative luciferase activity of pMIR-CDS1 c-Cbl (50% increase in T47D, 100% increase in MCF-7 and 90% increase in MDA-MB-231 cells) (Figure 2C, left panel). The miR-675 inhibitor had no effect on luciferase activity of the other constructs including pMIR-mut CDS1, pMIR-CDS2 or pMIR-3′UTR of c-Cbl. Similar results were obtained with Cbl-b plasmid (Figure 2C, right panel). The miR-675 inhibitor increased luciferase activity of only pMIR-CDS2 plasmid in the three tested cells lines, but had no effect on luciferase activity of the other constructs (Figure 2C, right panel).

Taken together, our results confirm that miR-675 decreases both c-Cbl and Cbl-b by interacting with coding sequence of theses mRNAs and precisely, with seed sequences predicted in the alignment.

### *H19*/miR-675 induce up-regulation of tyrosine kinase receptors and activation of the downstream AKT and ERK pathways

**Figure 3:**
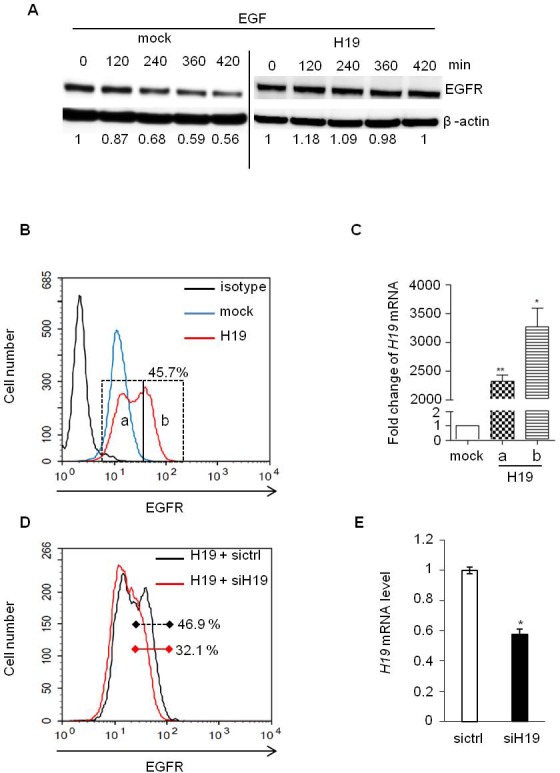
EGF-induced EGFR downregulation was prevented in *H19* overexpressing cells **A.** MDA-MB-231 control (mock) and *H19*-overexpressing cells (H19) were treated with EGF (50 ng/ml) and proteins were extracted at indicated times. Total EGFR levels were determined by western blot. The intensities of bands were quantified by densitometry (multigauge, Fujifilm), and the results obtained for EGFR expression during the time-course were compared to those obtained in control (indexed to 1) after normalization to actin expression. **B.** Flow cytometer analysis of membrane EGFR in MDA-MB-231 control cells (mock) and *H19*-overexpressing cells (H19). **C.** Subpopulations of *H19* overexpressing cells with medium and high expression levels of cell surface EGFR (peaks a and b in Figure 3B) were sorted by FACS, and *H19* expression was then quantified by qRT-PCR. Results are presented as relative levels in cells overexpressing *H19* compared to control. *p < 0.05; **p < 0.005. **D.** *H19*-overexpressing MDA-MB-231 cells were transiently transfected with siRNA-*H19*, and membrane EGFR levels were then determined by flow cytometry analysis. **E.** Relative expression of *H19* determined by qRT-PCR in breast cancer cells overexpressing *H19* transfected with siRNA-H19 or siRNA-GFP as a control. *p < 0.05.

c-Cbl and Cbl-b are well known to be involved in the degradation of tyrosine kinase receptors after their activation by growth factors [25]. To evaluate the role of *H19*/miR-675 in this process, starved MDA-MB-231 cells overexpressing or not *H19* were cultivated in the presence of EGF for different periods of time and then analyzed for the expression levels of EGF receptor (EGFR) by Western blot (Figure 3A). In control cells (mock), EGFR levels decreased progressively upon EGF treatment with nearly half (56%) of the initial EGFR expression present after 7h of culture in the presence of EGF (Figure 3A, left panel). By contrast, the expression levels of EGFR were not modified in cells overexpressing *H19* (Figure 3A, right panel). This suggests that *H19*/miR-675 induced-down regulation of c-Cbl and Cbl-b may contribute to impair the degradation of tyrosine kinase receptors. We then evaluated cell surface expression of EGFR by flow cytometer analysis (Figure 3B). Cells overexpressing *H19* clearly exhibited higher levels of cell surface EGFR compared to mock cells. Interestingly, in contrast to parental (not shown) and mock cells (Figure 3B), *H19*-overexpressing cells, exhibited two subpopulations expressing medium EGFR level (referred as peak a) and high EGFR level (referred as peak b) (Figure 3B). The two cell subpopulations were sorted by flow cytometry and analysed by qRT-PCR for *H19* expression (Figure 3C). Cells exhibiting a higher level of cell surface EGFR (peak b) also expressed higher *H19* expression levels as compared to peak a (Figure 3C) suggesting that the expression level of *H19* correlated with cell surface EGFR expression. To further investigate this relationship, we delivered siRNA to knock down *H19* expression in these *H19*-overexpressing MDA-MB-231 cells. Efficiency of siRNA was determined by qRT-PCR (Figure 3E). As shown in Figure 3D, transient *H19* siRNA expression decreased cell surface EGFR level, thus confirming the ability of *H19* to upregulate EGFR.

**Figure 4:**
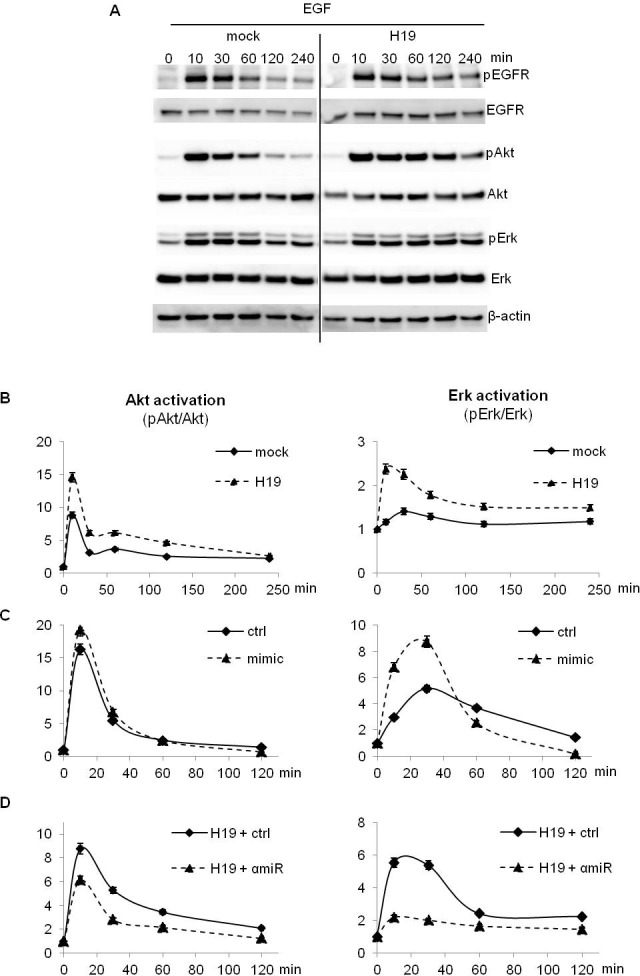
EGF-induced Akt and Erk phosphorylation was enhanced in *H19*/miR675-overexpressing cells **A.**, **B.** Control (mock) and *H19*-overexpressing cells (H19) were treated with 10 ng/ml EGF and proteins were extracted at indicated times. Akt and Erk activation was determined by Western blot analysis **A.** and ALPHAscreen^®^ **B.**. **C.** Parental MDA-MB-231 cells (ctrl) were transfected with miR-675 (mimic) for 72 h. Then, cells were treated with 10 ng/ml EGF. Proteins were extracted at indicated times and Akt and Erk activation was performed by ALPHAscreen^®^ analysis. **D.** *H19*-overexpressing cells (H19) were transfected with miR-675 specific inhibitor (αmiR) for 72 h. Then cells were treated with 10 ng/ml EGF. Proteins were extracted at indicated times and Akt and Erk activation was determined by ALPHAscreen^®^ analysis. These experiments were performed three times in triplicate. Data represent one representative experiment. For ALPHAscreen^®^ error bars represent SEM.

We then treated cells with EGF and studied activation of EGFR and its downstream signaling pathways over the time by Western blot and ALPHAscreen^®^. In MDA-MB-231 breast cancer cells stably overexpressing *H19*, EGF strongly activated EGFR, Akt and Erk compared to parental cells (Figure 4A and 4B). Interestingly, HGF treatment induced also a stronger activation of its receptor c-Met, Akt and Erk in MDA-MB-231 cells stably overexpressing *H19* compared to parental cells (Supplementary Figure 1A-1C). Similarly, *H19*-overexpressing cells exhibited stronger activation of Akt and Erk upon proNGF and NGF treatments (Supplementary Figure 1D and 1E). When cells were transiently transfected with a plasmid encoding the *H19* gene and then treated with EGF or HGF (Supplementary Figure 2), similar results were obtained in terms of Akt and Erk activation (Supplementary Figure 2).

To further verify if the increased activation of AKT and Erk was mediated by miR-675, we first transfected MDA-MB-231 parental cells with miR-675 mimic and cells overexpressing *H19* with the miR-675 inhibitor or the corresponding controls. Then, we treated cells with EGF and studied Akt and Erk phosphorylation. As shown in Figure 4C, cells transfected with mimic and then stimulated with EGF exhibited an increase of Akt and Erk activation compared to control vector transfected cells. Conversely, in *H19*-overexpressing cells, the miR-675 inhibitor decreased Akt and Erk activation (Figure 4D).

All together, our results indicate that *H19*/mir-675-5p increase the activation of several tyrosine kinase receptors and their downstream signaling pathways in breast cancer cells.

### *H19*/miR-675 increase migration and proliferation of breast cancer cells and enhance the effects of growth factors

**Figure 5:**
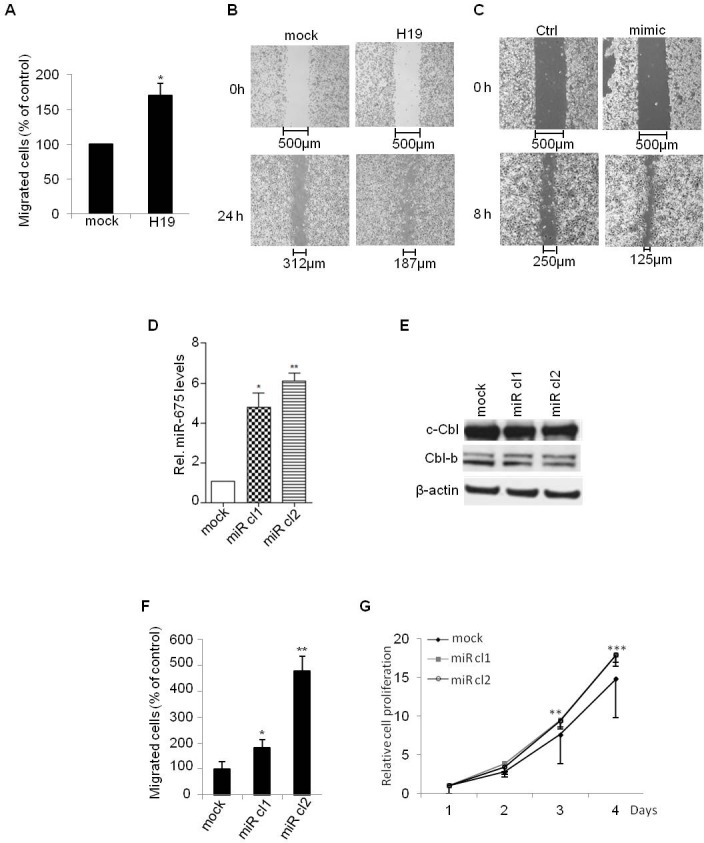
*H19*/miR675 increased migration and proliferation of MDA-MB-231 breast cancer cells **A.** Control (mock) or *H19-*overexpressing (H19) cells were cultured in transwells for 24 h. Migrated cells were then colored with violet crystal and counted. Results are presented as the percentage of control. **B.** Wound healing assay performed on control (mock) and *H19-*overexpressing (H19) cells. **C.** Wound healing assay performed on control (ctrl) and transiently overexpressing miR-675 (mimic) cells. **D.** QRT-PCR analysis of expression of miR-675-5p in control (mock) and miR-675-overexpressing cells (miR cl1, miR cl2). Results are presented as relative levels compared to MDA-MB-231 control cells (indexed to 1). **E.** Western blot analysis of c-Cbl and Cbl-b levels in control and miR-675-overexpressing cells. **F.** Transwell migratory assay performed on control (mock) and miR-675-overexpressing cells after 6 h of culture. **G.** Cell proliferation determined by MTT test. Data represent mean of three independent experiments and error bar sem.*p < 0.05; **p < 0.005; ***p < 0.001.

To further determine the functional impact of Akt and Erk activation in cells overexpressing *H19* and miR-675, we first evaluated the migratory capacity of *H19*-overexpressing cells using transwell and wound-healing assays. As shown in Figure 5A and 5B, *H19*-overexpressing MDA-MB-231 cells exhibited an increased migratory capacity compared to control cells. Moreover, *H19* overexpression further increased HGF-induced migration of MDA-MB-231 cells (Supplementary Figure 3A). Similarly, *H19* gene overexpression increased also EGF-induced proliferation of MCF-7 cells (Supplementary Figure 3B).

To evaluate the role of miR-675 independently of *H19* mRNA, we first evaluated the migratory ability of parental MDA-MB-231 transiently transfected with miR-675 mimic. We found that miR-675 mimic increased cell migration as revealed by wound healing assay (Figure 5C). This indicates that miR-675 *per se* is able to increase cell mobility. We then generated cells stably overexpressing miR-675. For this, we introduced in MDA-MB-231 cells a plasmid encoding Green Fluorescent Protein (GFP) fused with miR-675 precursor sequence. As shown in Figure 5D, the two selected clones (miR cl1 and miR cl2) expressed more than 5-fold of miR-675 when compared to mock cells. As expected, the protein levels of c-Cbl and Cbl-b were decreased in the two selected clones (Figure 5E). Importantly, these clones exhibited stronger migratory and proliferative capacities when compared to control cells (Figure 5F and 5G). Together, these data confirm that miR-675 increases cell migration and proliferation independently of *H19*.

### The miR-675 enhances the tumorigenicity and metastatic potential of breast cancer cells

**Figure 6:**
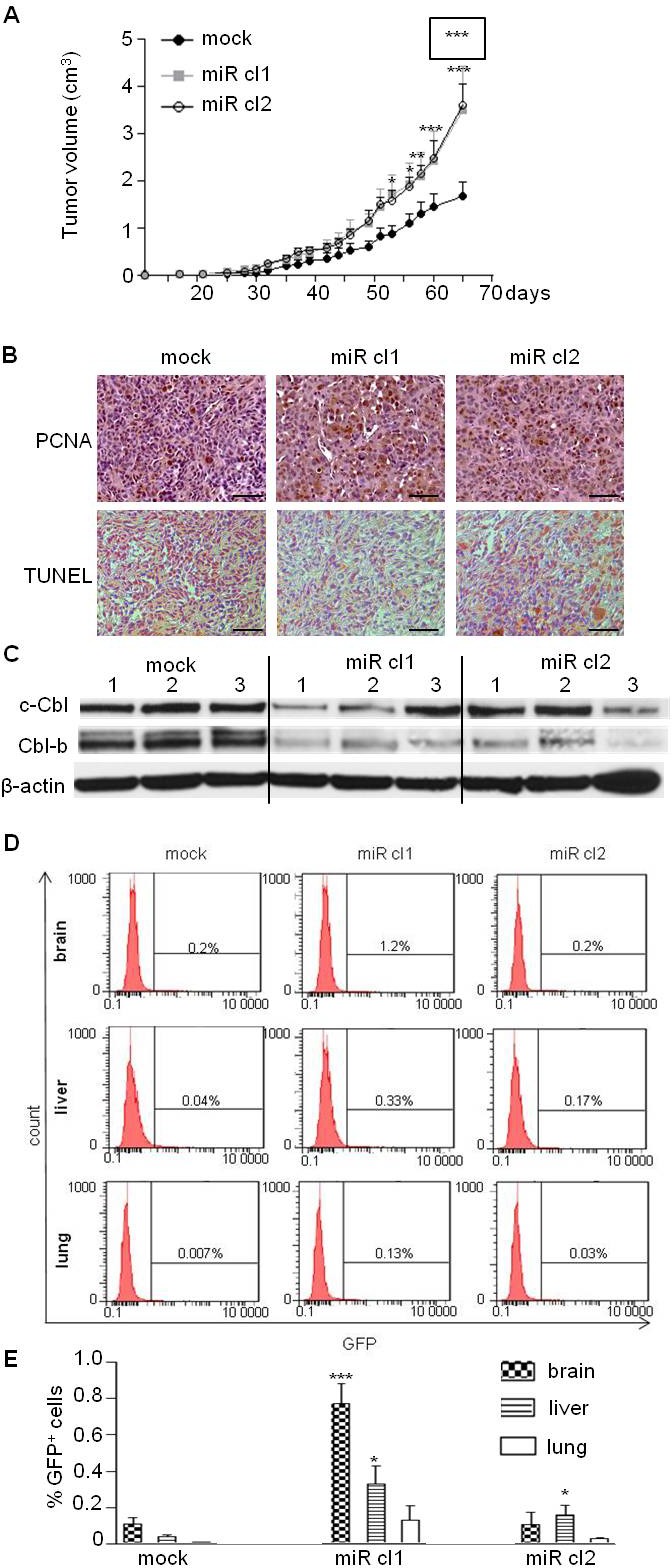
MiR-675 promoted tumor growth and metastasis **A.** MDA-MB-231 control (mock) and overexpressing miR-675 (miR cl1, miR cl2) cells were subcutaneously injected into SCID mice (7 mice per group). Tumor growth curve represents the mean of tumor volumes in each group and error bar sem. MiR cl1 growth curve statistical analysis is surrounded. **B.** Detection of tumor cell proliferation (PCNA) and apoptosis (TUNEL) of paraffin-embedded sections of tumors 65 days after injections. Scale bar is 100μm. **C.** Western blot analysis of c-Cbl and Cbl-b levels in xenografted tumors. Tumors from three mice for each group were analyzed (mock, miR cl1, miR cl2). **D.** and **E.** Detection of GFP-positive cells by flow cytometry analysis in brain, liver and lung of mice xenografted with control or miR-675-overexpressing cells (mock, miR cl1, miR cl2). Histograms in E represent the mean of results from six xenografted mice *p < 0.05; **p < 0.005; ***p < 0.001.

Since ectopic expression of miR-675 increased migration and proliferation of breast cancer cells *in vitro,* we examined whether overexpression of miR-675 could enhance tumor growth and metastasis *in vivo* by subcutaneously injecting cells into immunodeficient SCID mice. As shown in Figure 6A, tumor volume was increased in mice injected with miR-675-overexpressing clones as compared to control mice injected with parental cells. Accordingly, an increased cell proliferation (PCNA staining) and a decreased apoptosis (TUNEL detection) were found in sections of tumors formed by miR-675-overexpressing cells (Figure 6B, middle and right panels) as compared to those formed by parental cells (Figure 6B, left panels). Of note, the protein levels of c-Cbl and Cbl-b were decreased in tumor formed by miR-675-overexpressing cells compared to control (Figure 6C). We then analyzed metastases in xenografted mice thanks to GFP expression in MDA-MB-231 cells. As shown in Figure 6D and 6E, more GFP-positive cells were found in brain, liver and lungs of mice xenografted with miR-675 overexpressing cells compared to control (Figure 6D, 6E). Taken together, these results indicate that miR-675 increases primary tumor formation and promotes metastasis.

## DISCUSSION/CONCLUSION

It has become increasingly clear that *H19* RNA plays essential role in tumor development and that the *H19* gene is regulated by a complex interplay of both extrinsic and intrinsic factors. We have already demonstrated that *H19* expression is positively regulated by several growth factors such as HGF, EGF and FGF-2 [21]. HGF induces *H19* expression *via* the ERK/MAPK and phospholipase C pathways. Here, we demonstrated that *H19* induced up-regulation of tyrosine kinase receptors including EGFR and c-Met as well as the activation of their downstream Akt and ERK signaling pathways. Our findings indicate that the positive feedback between *H19* expression and growth factors may be of importance in promoting breast cancer development.

More recently, *H19* has been found to encode miR-675. Several targets of miR-675 have been identified in cancers from different tissues. These include Twist1, CALN1, TGFβ1, and Cadherin11 [13, 18, 26, 27]. While this study was underway, it was shown that miR-675 regulates negatively Rb expression by interacting with its 3′UTR mRNA in colorectal cancer and hepatocellular carcinoma [12, 18]. However, we did not find any regulation of Rb by miR-675 in breast cancer cells (Supplementary Figure 4). Our results together with the previous identification of miR-675 targets indicate that miR-675 may function in a tissue-specific manner and that the increased proliferation of *H19*-overexpressing breast cancer cells cannot be explained by a negative regulation of Rb. Indeed, we demonstrated that miR-675 decreased the expression of c-Cbl and Cbl-b. MiR-675 interacted directly with c-Cbl and Cbl-b coding sequences to prevent mRNA translation. Among the reported targets of miR-675, c-Cbl and Cbl-b were the first ones found to interact with miR-675 *via* their coding sequences.

In mammals, three homolog of Cbl exist: c-Cbl, Cbl-b and Cbl-c. The protein structures of c-Cbl and Cbl-b are similar; they consist of a N-terminal tyrosine kinase-binding domain, a ring finger motif, a proline-rich region and a c-terminal ubiquitin-associated domain that overlap leucine zipper motif. The Cbl-c lacks the c-terminal domain (proline-rich region and ubiquitin-associated domain). Cbl proteins are involved in the regulation of actin skeleton, lymphocyte signaling and downregulation of tyrosine kinase receptors [28]. Cbl proteins negatively regulate tyrosine kinase receptors by interacting with Grb2 to prevent Grb2/SOS association. Cbl can also recognize activated receptors and induce their downregulation by lysosomal degradation.

In breast cancer cells, it has been demonstrated that c-Cbl is associated with two other proteins to form the tripartite complex Cdc42, p85Cool-1/βPix, and c-Cbl [29]. Upon EGFR activation, Cdc42 interacts with c-Cbl within the tripartite complex to repress the degradation of the activated receptor initiated by c-Cbl. In lung cancer cells, c-Cbl inactivating-mutation increases viability and migration of cells [30]. Similarly, in acute myeloid leukemia, c-Cbl mutations decrease EGFR ubiquitinylation, leading to signaling activation, cell proliferation and survival. Moreover, c-Cbl has been proposed to act as a tumor suppressor since c-Cbl null mice develop invasive cancer (notably, juvenile myelomonocytic leukemia) with complete penetrance [31]. In accordance with these data, we demonstrated that miR-675-induced decrease of c-Cbl and Cbl-b was associated with a more aggressive phenotype of breast cancer cells; miR-675-overexpressing cells presented increased levels of EGFR and c-Met (not shown), increased activation of the downstream signaling pathways as well as increased tumor growth and metastasis. In agreement with our data, Matouk et al demonstrated that miR-675 indirectly targets slug leading to increase of cell invasion and *in vivo* metastasis [32].

On the other hand, Berberine (an isoquinoline alkaloids used in gastroenteritis, type II diabetes, hypertension and arrhythmia) inhibits proliferation of colon cancer cells by stimulating c-Cbl activation and enhancing EGFR degradation [33]. Similarly, another drug, icotinib, activates Cbl-b protein to downregulate EGFR, and induces apoptosis and G1 phase arrest in non-small-cell lung cancer cells [34]. Thus, cancer therapeutic agents can induce downregulation of tyrosine kinase receptors by activating Cbl proteins. However, cancer resistance to chemotherapy is also frequently associated with the overexpression of tyrosine kinase receptors, which can serve as a link between tumor cells and the microenvironment [35]. The activation of tyrosine kinase receptors by specific growth factors in tumor microenvironment can protect tumors cells from drugs-induced damages. Our findings suggest that drug resistance of *H19*-overexpressing cancer cells could be due to Cbl downregulation by *H19*-derived miR-675. Supporting this hypothesis, we found that high expression of miR-675 combined with low expression of c-Cbl identified a subset of ER-negative breast cancer tumors of poorer prognosis (Supplementary Figure 5). This was found by exploiting the matched mRNA and microRNA global expression profiling of a cohort of 207 primary tumors [36] and, given the rarity of such cohort, will need to be confirmed by future similar studies.

An antisense transcript, *91H*, has been found in the *H19* locus [37, 39]. We verified if this long non coding RNA can act as a miR sponge and impair miR-675 effects. *91H* expression does not vary in miR-675 overexpressing cells. In addition, in *91H* knockdown cells, c-Cbl and Cbl-b expression is stable (Supplementary Figure 6), indicating *91H* is not involved in c-Cbl and Cbl-b regulation.

In conclusion, we identified ubiquitin ligase E3 family (c-Cbl and Cbl-b) as direct targets of miR-675 in breast cancer cells. Moreover, by directly targeting c-Cbl and Cbl-b, miR-675 increased the stability and the activation of EGFR and c-Met leading to subsequent activation of Akt and Erk as well as enhanced cell proliferation and migration. Our findings provide novel mechanistic insights into a critical role for *H19* RNA in breast cancer development, and reveal a link between *H19*/miR-675, Cbl and tyrosine kinase receptors to enhance breast cancer cell aggressiveness.

## MATERIALS AND METHODS

### Cells culture

The MCF-7 and T47D estrogen-sensitive and the MDA-MB-231 estrogen-insensitive breast cancer cell lines were obtained from the American Type Culture Collection and maintained routinely in *Roswell Park Memorial Institute medium* (RPMI, Gibco) containing 10% of foetal bovine serum (FBS) and 0.01% of Zell Shield (Minerva Biolabs). Cell lines were cultured at 37°C with 5% CO_2_, 95% of air in humidified atmosphere.

### Migration assays

Cell migration was determined by transwell assay and wound healing assay. For transwell assay, 3×10^4^ cells were seeded on collagen (1/100, Millipore) coated insert (0.8μm, BD Bioscience) of 6 well-plates in RPMI containing 10% FBS or 0.1 % FBS in the presence of HGF. At the end of the experiments, cells migrating to the other side of the filter were stained with 0.5% crystal violet and counted. Wound healing test was performed by using Culture-inserts for Live Cell Analysis (Ibidi). A total of 3×10^4^ cells were plated on each compartment of insert and cultured for 5h. The insert was then removed (0h) and cells were further cultured for 24h. Cells were photographed at 0h and after 24h of culture to record the wound width.

### Proliferation assays

A total of 500 cells were plated in each well of a 96-well plate. Cell proliferation was determined using MTT test every day as previously described [12].

### Western blot analysis

Cells were lysed in RIPA buffer containing protease and phosphatase inhibitors (protease inhibitor P8340; phosphatase inhibitor cocktail 2 P5726, Sigma-Aldrich). Proteins were quantified with BCA protein assay (Pierce) and then reduced in NuPAGE LDS Sample buffer (Invitrogen) with NuPAGE Reducing Agent (Invitrogen) at 70°C for 10 min. Proteins were separated on SDS-PAGE 4-12% (Invitrogen) and transferred onto PolyVinylidene Fluoride (PVDF) membrane (Millipore). After saturation in PBS 0.2% of casein, membranes were incubated with primary antibodies overnight at 4°C. References of antibodies are listed in the supplemental Table S1. Membranes were washed with PBS 0.5% Tween for 30 min and incubated with secondary antibodies conjugated with Horse Radish Peroxidase (HRP) for 2 h at room temperature. Membranes were analysed with SuperSignal west Dura Chemiluminescence Substrate (Pierce).

### Quantification of protein phosphorylation by ALPHAscreen^®^

Quantification of protein phosphorylation was performed using ALPHAscreen^®^ SureFire technology (Perkin Elmer). This technology allows protein activation quantification in low volume of lysates. Briefly, cells were lysed in appropriate buffer completed with protease and phosphatase inhibitors and lysates were clarified by centrifugation. Lysates were incubated with different antibodies and acceptor beads for 2 h at room temperature on 384-well plates. Then donor beads were added for 2 h and light emission was measured on EnSpire Alpha (Perkin Elmer). The anti-p-Erk Thr202/Tyr204 (TGRESHV100), anti-total Erk TGRTES500, anti-p-Akt ser473 (TGRA4S500), anti-total Akt (TGRTAPS500), anti-p-Met Tyr1234/1235 (TGRCMS500), and anti-total Met (AL281C) antibodies were from Perkin Elmer.

### RNA extraction, reverse transcription and Real-time RT-PCR

RNA extraction and qRT-PCR was performed as previously described [37]. Primers used for qRT-PCR are described in supplemental Table S2.

MiRNA was extracted with mirVANA^TM^ miRNA isolation kit (Life Technologies). For the detection, a total of 50 ng RNA was used in the reverse transcription reaction (miRCURY LNA^TM^ Universal RT) (Exiqon). Quantitative PCR was performed by using Exilent SYBR Green master mix (Exiqon) and hsa-miR-675-5p LNA PCR primer set, UniRT (Exiqon). Human U6 RNA was used as an internal control. Fold change of miRNA expression was calculated by the equation 2^−ΔΔCt^.

### Construction of plasmids

Coding DNA Sequence (CDS) and 3′UTR of c-Cbl and Cbl-b mRNA were cloned in the pMIR-REPORT luciferase vector (Ambion). Because of their length, CDS were cloned in two fragments named CDS1 and CDS2. All fragments of Cbl-b and 3′UTR of c-Cbl were cloned between *Spe*I and *Hin*dIII enzyme site. CDS1 of c-Cbl was cloned between *Spe*I and *Pme*I and CDS2 between *Spe*I and *Mlu*I (New England Biolabs). Fragments were generated by PCR (primers are listed in supplemental Table S2). Point mutations in seed sequence were generated by PCR.

*H19* gene was cloned in pcDNA3.1 (−) (Invitrogen) between *Not*I and *Bam*HI (New England Biolabs). The normalizing vector pRL-null has no promoter sequence to drive expression of the *Renilla* luciferase gene and was purchased from Promega.

MiR-675-5p was amplified by PCR and cloned in pEGFP-C1 plasmid (Clontech) between *Hin*dIII and *Bam*HI (New England Biolabs). Plasmid productions were performed in *E. Coli* TOP 10 (Invitrogen). Plasmid extraction was performed by using Nucleobond PC100 (Macherey-Nagel).

### H19 gene and siH19 transient transfection

For transient transfection, a total of 1.3 × 10^5^ cells were plated on 6-well plates. After 24h, cells were transfected with 1μg of pcDNA3.1-*H19* DNA using Exgen-500 (Euromedex) according to the manufacturer's instructions. Cells were incubated at 37°C for 6h, and then cultured in medium used routinely. *H19* siRNA (Table S2) were transfected with DharmaFECT Duo according to manufacturer's guidelines (Thermo Fischer Scientific Dharmacon).

### Establishment of cells overexpressing H19 or miR675

To establish cells overexpressing *H19*, MDA-MB-231 and MCF-7 breast cancer cell lines were transfected with 1μg of pcDNA3.1-H19 or pcDNA3.1 empty vector as a control using Exgen-500 and cells were allowed to recover for 48h. Cells were then selected in the presence of 1mg/ml G418 (Sigma) for at least one month. To establish cells overexpressing miR675, MDA-MB-231 cells were transfected with 1 μg of pEGFP-C1 /miR675 or pEGFP-C1 empty vector using Exgen-500 for 48h. Cells were then selected in the presence of 1mg/ml G418 (Sigma) for at least one month. Among numerous clones obtained, two of them were randomly chosen for *in vitro* and *in vivo* experiments.

### MicroRNA transfection and luciferase activity assay

A total of 1 × 10^5^ cells were plated on 12-well plates for 24h. Hsa-miR-675 mimic or its hairpin inhibitor (Thermo Fischer Scientific Dharmacon) were transfected with DharmaFECT-Duo according to manufacturer's guidelines (Thermo Fischer Scientific Dharmacon). Briefly, 75 nM mimic or hairpin inhibitor (anti-miR) were transfected with 500 ng of *Firefly* luciferase plasmid and 12 ng of pRL-null plasmid expressing Renilla luciferase to monitor the transfection efficiency. The luciferase activity was measured 24h after transfection by using the dual-luciferase reporter assay system according to manufacturer's instructions (Promega). *Firefly* luciferase was normalized with *Renilla* luciferase.

### Flow cytometer analysis of membrane EGFR

A total of 2 × 10^5^ cells were plated on 6 well-plates and cultured in RPMI containing 1% FBS. After 48h, cells were isolated by trypsinization using trypsin-versene EDTA solution, then incubated with an anti-EGFR antibody (EGFR-AF488, 1/100, SC-120, Santa Cruz) for 30 min at 4°C before analysis on flow cytometer (Calibur II or ARIA II, Becton Dickinson).

### Tumorigenesis in SCID mice

Female SCID mice (8-weeks-old) were purchased from Pasteur Institute, Lille and kept under pathogen-free condition. Animals were handled in accordance with the European Communities Recommendations for Animals Experimentation. Exponentially growing cells were harvested, resuspended in PBS, and subcutaneously (2 × 10^6^ cells in 150 μl) injected into the flank fat pad of each mouse. Tumor volume and metastasis were monitored as previously described [38].

### Analysis of cell proliferation and apoptosis in xenografted tumors

Tumors were fixed, paraffin embedded and cut on 5 μm tumor sections. Cell proliferation was measured by immunostaining with an anti-PCNA antibody (Santa Cruz, sc-56 HRP). Apoptosis was measured using *in situ* cell death detection kit, POD (Roche), according to manufacturer's instructions.

### Statistical analysis

Data are expressed as mean values ± standard error of the mean of at least 3 independent experiments. The statistical analysis was done by using Student's *t*-test and p value < 0.05 was considered significant.

## SUPPLEMENTARY MATERIAL FIGURES AND TABLES


