# Supplementary material for: H19 non coding RNA-derived miR-675 enhances tumorigenesis and metastasis of breast cancer cells by downregulating c-Cbl and Cbl-b
Source: Oncotarget. 2015 Jul 22;6(30):29209–23. doi: 10.18632/oncotarget.4976 (PMC4745721; doi:10.18632/oncotarget.4976)
Supplement: Supplementary file 1 [file oncotarget-06-29209-s001.pdf]

H19 non coding RNA-derived miR-675 enhances tumorigenesis and metastasis of breast cancer cells by downregulating c-Cbl and Cbl-b

SUPPLEMENTARY MATERIAL

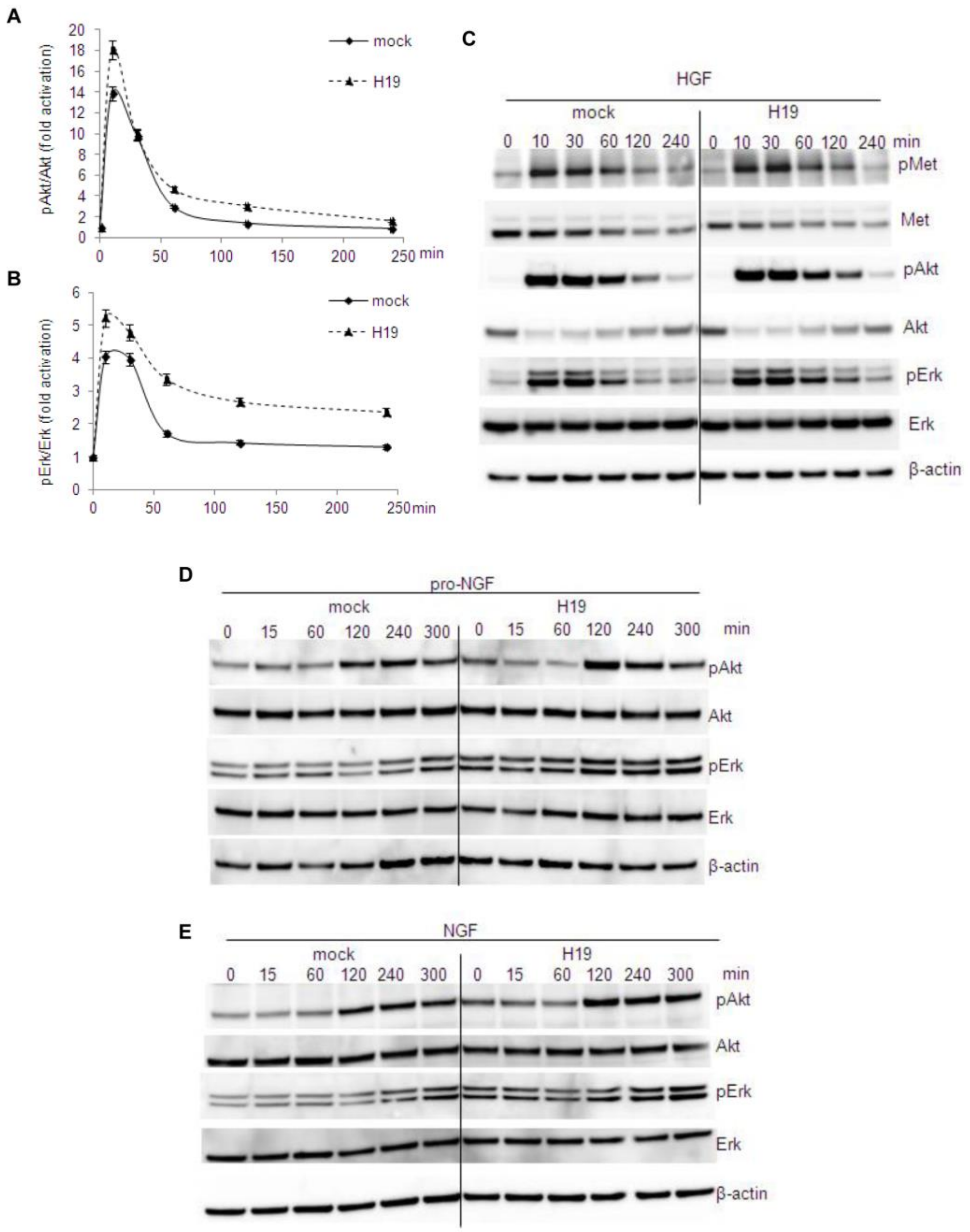

Supplementary Figure 1. Growth factors induced Akt and Erk phosphorylation in MDA-MB-231 cell stably overexpressing *H19*. (**A, B, C**) Control (mock) and *H19* overexpressing cells (H19) were treated with 10 ng/ml HGF. Proteins were extracted at

indicated times and Met, Akt and Erk activation was determined by ALPHAscreen® (**A, B**) and Western blot analysis (**C**). (**D**, **E**) Cells were treated with 10 ng/ml pro-NGF (**D**) or 100 ng/ml NGF (**E**). Proteins were extracted at indicated time and Akt and Erk activation were determined by Western blot analysis. These experiments were performed three times in triplicate. Data represent one representative experiment. For ALPHAscreen® error bars represent the SEM.

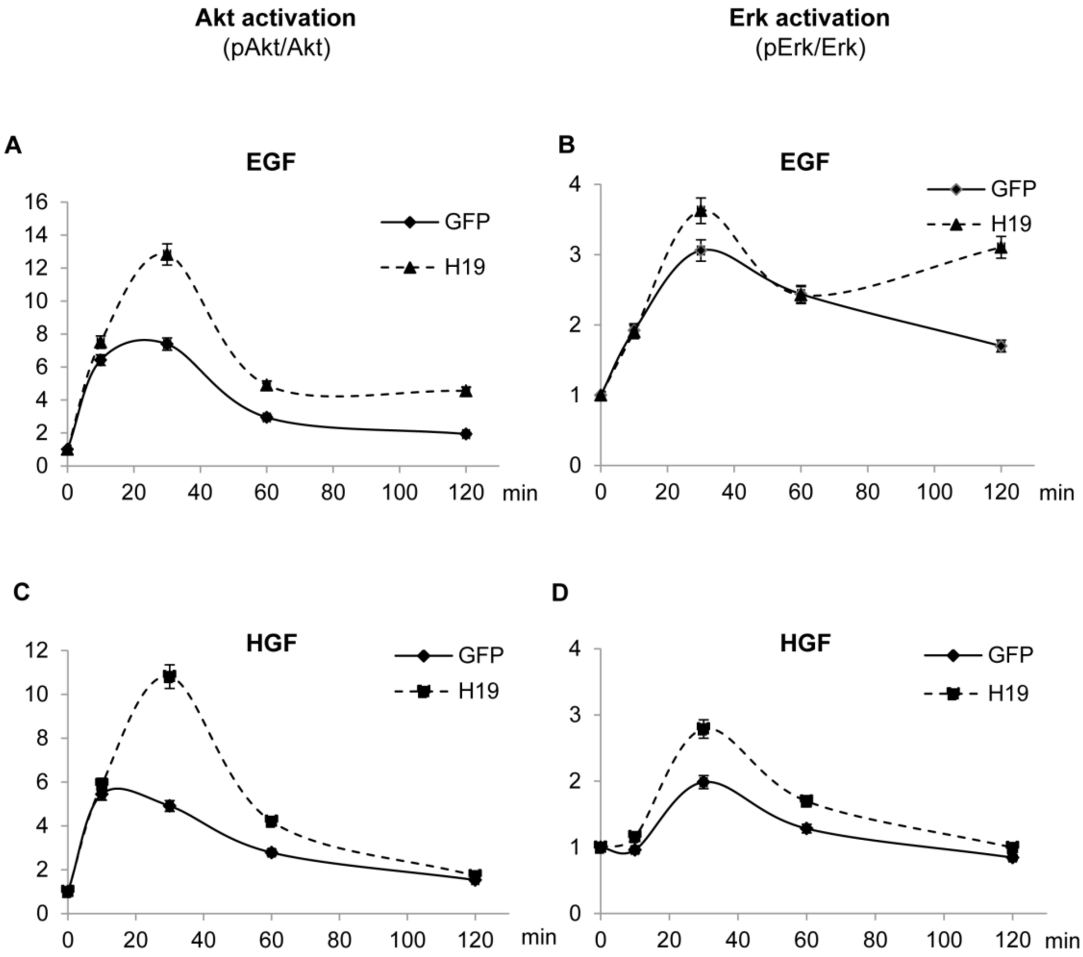

Supplementary Figure 2. HGF and EGF induced Akt and Erk phosphorylation in MDA-MB-231 cell transiently overexpressing *H19*. Cells transiently transfected with GFP plasmid or *H19* plasmid for 72 H were treated with 10 ng/ml EGF (**A**, **B**) or 10 ng/ml HGF (**C**, **D**). Proteins were extracted at indicated times and activation of Akt and Erk was determined by ALPHAscreen®. These experiments were performed three times in triplicate. Data represent one representative experiment and error bars the SEM.

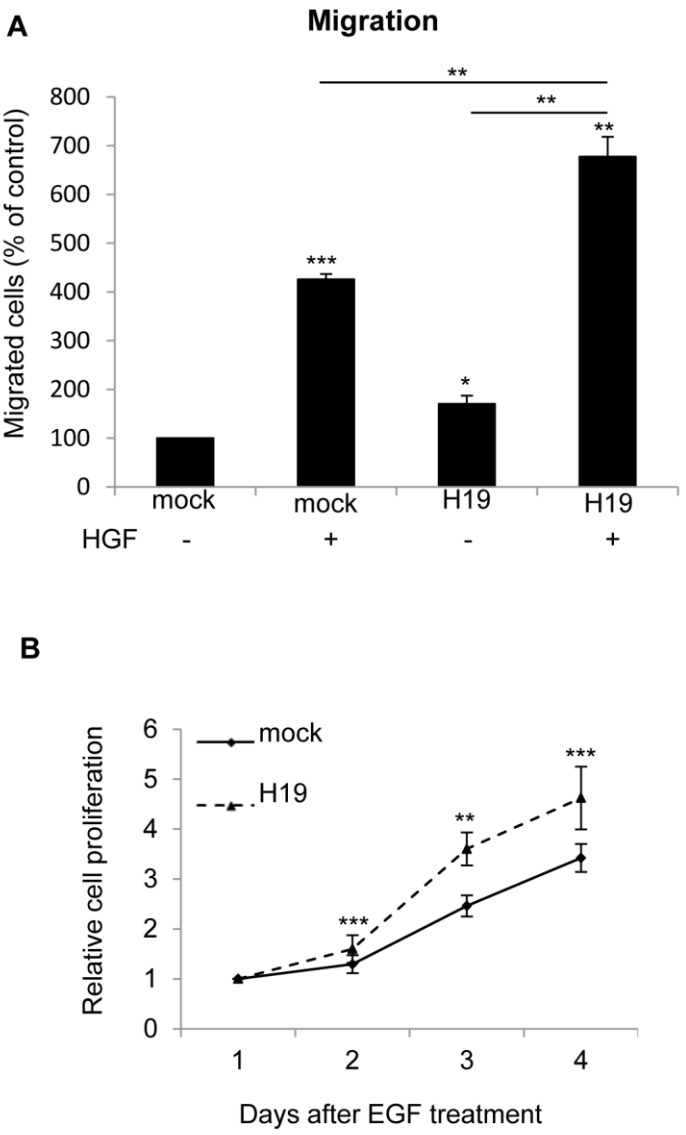

Supplementary Figure 3. ***H19* increased migration and proliferation of breast cancer cells. (A)** Control (mock) or *H19*-overexpressing (H19) MDA-MB-231 cells were cultured in transwells in the presence or absence of 10 ng/ml HGF for 24 h. Migrated cells were then colored with violet crystal and counted. Results are presented as the percentage of non-treated control cells. (**B**) Control (mock) and *H19*-overexpressing (H19) MCF-7 cells were cultured in the presence of 10 ng/ml EGF. Cell proliferation was determined by MTT test at indicated days. *p<0.005; **p<0.005; ***p<0.001

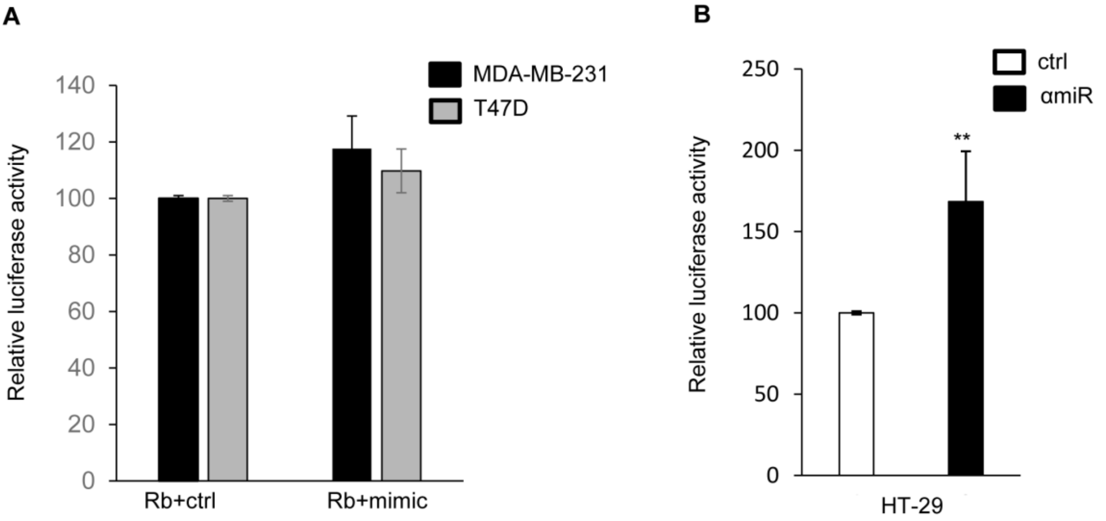

Supplementary Figure 4. Rb is not regulated by *H19* in breast cancer cells. The firefly luciferase activity in breast **(A)** and colorectal **(B)** cancer cells after cotransfection with reporter construct and miR-675 mimic or miR-675 inhibitor (αmiR). The luciferase activity was measured by dual-luciferase reporter assay (Promega) and was normalized to Renilla luciferase activity. Plasmids were transfected with mimic or antimir, or theirs controls in breast and colorectal cancer cells lines (MDA-MB-231, T47D and HT-29). Data represent mean of three independent experiments *versus* their respective controls in percentage and error bar, sem. **$p<0.01$

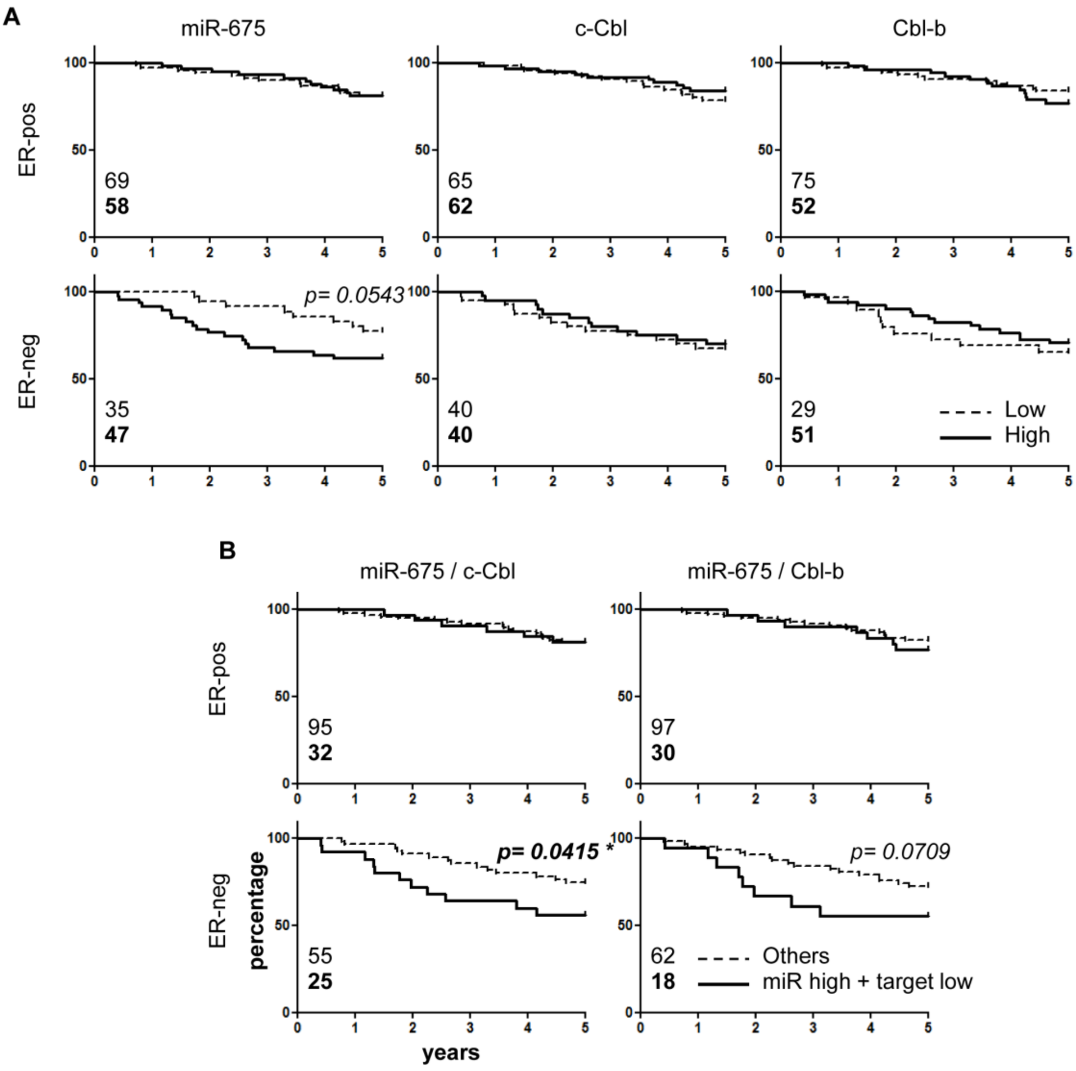

Supplementary Figure 5. MiR-675 expression is associated to poor free survival. microRNA and mRNA expression data were collected from a previously published cohort [36], NCBI accession number GSE22220. We used Graphpad Prism to analyze the data corresponding to miR-675 (probe ILMN_3167407), c-Cbl (probe 1230102) and Cbl-b (probe 4010563). Tumors were classified as 'low' when ≤ to the median and 'high' otherwise. (**A**) Kaplan-Meïer analyses were performed for each marker individually after ER stratification. (**B**) Kaplan-Meïer analyses were performed by comparing the patients expressing both a high level of miR-675 and a low level of its putative target (c-Cbl or Cbl-b). Number of patients included is indicated next to each curve. P values were calculated using Grehan-Breslow–Wilcoxon test.

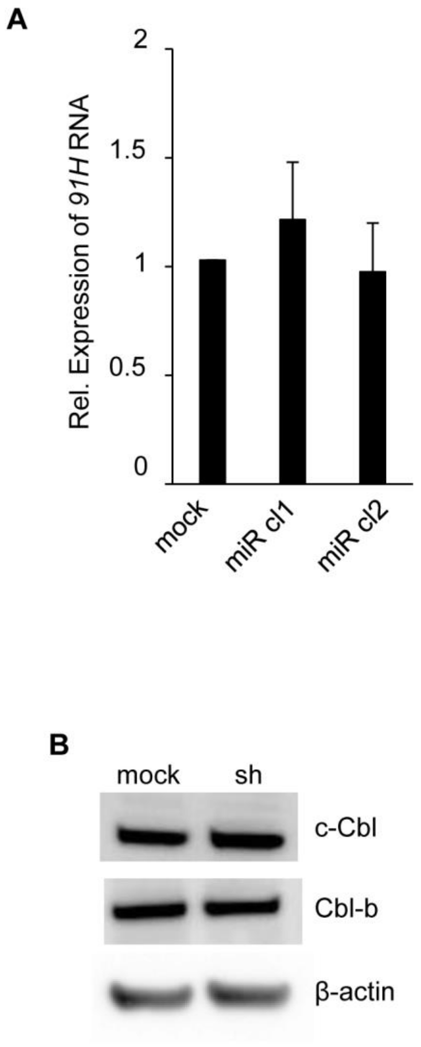

Supplementary Figure 6. ***91H*** **RNA is not a sponge of miR-675.** (**A**) Relative expression of *91H* in miR-675 overexpressing cells (miR cl1, miR cl2) determined by qRT-PCR. Data represent mean of three independent experiments and error bar, sem. (**B**) Western blot analysis of c-Cbl and Cbl-b in breast cancer cells, MDA-MB-231, transfected with *91H* shRNA (sh) compared to control cells (mock).

Supplementary Table 1. Antibodies used for western blot analysis.

Antibody	Reference	Origin
p-Met	#3135	Cell signaling
Met	37-0100	Invitrogen
p-EGFR	#1138-1	Epitomics
EGFR	AHR5062	Invitrogen
p-Akt	4060S	Cell signaling
Akt	2920	Cell signaling
p-Erk	#9106	Cell signaling
Erk	C14/sc-154	Santa Cruz
c-Cbl	Sc-170	Santa Cruz
Cbl-b	Sc-8006	Santa Cruz
βactin	Sc-47778	Santa Cruz

Supplementary Table 2. Oligonucleotides used for plasmid construction. Primer used for qRT-PCR. Sequence of *H19* siRNA.

Primer	5' to 3'
	**cloning**
c-Cbl CDS1 SpeI	AACTAGTGGCCGGCAACGTGAAGAAGAGCT
c-Cbl CDS1 PmeI	CGTTTAAACGCTGCCACTCCCTCTAGGATC
c-Cbl CDS2 SpeI	GACTAGTGAGGGAGTGGCAGCCTGTTGAGG
c-Cbl CDS2 MluI	ACGCGTCTAGGTAGCTACTAGGGCAGG
c-Cbl 3'UT SpeI	TACTAGTCACACCATCTCCCTGCTGCAGGT
c-Cbl 3'UT HindIII	AAGCTTCACCTTCAAATGCACTCAAGA
Mut c-Cbl antisense	TGGACGTTAGGCTGGAAGGCGTCCTTCATG
Mut c-Cbl sense	CATGAAGGACGCCTTCCAGCCTAACGTCCA
Cbl-b CDS1 SpeI	CACTAGTGCAAACTCAATGAATGGCAGAAA
Cbl-b CDS1 HindIII	TAAGCTTCGTCCAAGTCTAGCATCGGCATG
Cbl-b CDS2 SpeI	CACTAGTATGCTAGACTTGGACGACGATGA
Cbl-b CDS2 HindIII	CAAGCTTCTATAGATTTAGACGTGGGGATA
Cbl-b 3'UTR SpeI	TACTAGTCAGCCAGAACTGTAGACACCAAA
Cbl-b 3'UTR HindIII	TAAGCTTATGGAAAACCCCTTACAAAAGG
Mut Cbl-b antisense	AGGACGTTATGGGAGTGGTTTTATCTTGTTT
Mut Cbl-b sense	AAACAAGATAAACCACTCCCATAACGTCCT
H19 FL 5p	AGCAGGGTGAGGGAGGGGGT
H19 FL 3p	GTAACAGTGTTTATTGATG
miR-675 HindIII	AAAGCTTAACGAGGCACTGCGGCCCAGGGT
miR-675 BamHI	AGGATCCTCTGCCAAGCCAGCCCCAGGGGC
	**RT-qPCR**
RPLP0 sense	GTGATGTGCAGCTGATCAAGACT
RPLP0 antisense	GATGACCAGCCCAAAGGAGA
H19 sense	TACAACCACTGCACTACCTG
H19 antisense	TGGCCATGAAGATGGAGTCG
c-Cbl sense	GTGATCCCTGGACAGGAAGA

c-Cbl antisense	GGTGGTCACACTCTGGACCT
Cbl-b sense	GAAGAAAGCCACAGCCTGAC
Cbl-b antisense	GGATTGGTGGAGGTCTTTCA
91H sense	GCTTGTAGTAGAGTGCGCC
91H antisense	CATCCAGTTGACCGAGCTTG
	**siRNA**
siRNA H19 1 sense	UAAGGUGUUCAGGAAGGCC
siRNA H19 1 antisense	GGCCUUCCUGAACACCUUA
siRNA H19 2 sense	AGCUUCACCUUCCAGAGCC
siRNA H19 2 antisense	GGCUCUGGAAGGUGAAGCU
